# The Growth Suppression Activity of Diosmin and PGV-1 Co-Treatment on 4T1 Breast Cancer Targets Mitotic Regulatory Proteins

**DOI:** 10.31557/APJCP.2021.22.9.2929

**Published:** 2021-09

**Authors:** Hajidah Musyayyadah, Febri Wulandari, Ana Fiin Nangimi, Afivah Dewi Anggraeni, Muthi’ Ikawati, Edy Meiyanto

**Affiliations:** 1 *Study Program of Biotechnology, Graduate School, Universitas Gadjah Mada, Yogyakarta, Indonesia. *; 2 *Cancer Chemoprevention Research Center, Faculty of Pharmacy, Universitas Gadjah Mada, Yogyakarta, Indonesia. *; 3 *Laboratory of Macromolecular Engineering, Department of Pharmaceutical Chemistry, Faculty of Pharmacy, Universitas Gadjah Mada, Yogyakarta, Indonesia.*

**Keywords:** Diosmin and PGV-1 co-treatment, 4T1- mitotic catastrophe, mitotic regulatory proteins, senescence

## Abstract

**Objective:**

We aim to enhance the effectiveness of curcumin analog PGV-1 through co-treatment with diosmin, a citrus flavonoid, on 4T1 cells and evaluate the molecular targets underlying its effect on the cell cycle.

**Methods:**

Cytotoxic effects were performed by MTT assay against 4T1 cells. The May Grünwald-Giemsa staining was used to observe cell cycle arrest. The senescence was assayed with SA-ß-gal staining. Bioinformatic studies were utilized to discover protein targets of PGV-1 and diosmin on triple-negative breast cancer (TNBC) using SwissTargetPrediction, then exploration of protein targets was performed using the TCGA dataset via the UALCAN website. Kaplan-Meier was performed using GraphPad with data from the TCGA dataset via Oncoln. Using MOE 2010, we conducted the binding affinity between PGV-1 and diosmin to protein targets.

**Results:**

PGV-1 and diosmin showed cytotoxic effect with IC_50_ values of 9 µM and 389 µM, respectively, and the combined cytotoxic assay exhibited a synergistic effect with a combination index (CI) of <1. PGV-arrested 4T1 cells in pro-metaphase and induced mitotic catastrophe, while the combination of diosmin with PGV-1 increased the number of mitotic catastrophes. The SA-ß-gal assay revealed that both compounds were capable of inducing senescence in 4T1 cells. Study bioinformatics and molecular docking showed that PGV-1 and diosmin target cell cycle regulatory proteins in TNBC, namely CDK1, KIF11, and AURKA. Thus, the combination of diosmin and PGV-1 modulating the cell cycle that causes senescence and catastrophic death of 4T1 cancer cells is related to the inhibition of these cell cycle proteins.

**Conclusion:**

Diosmin enhances the cytotoxic effect of PGV-1 synergistically on 4T1 cancer cells, which correlates to the increasing senescence and mitotic catastrophe. The synergistic effect of the co-treatment is likely to target AURKA, CDK1, and KIF11. The combination of PGV-1 and diosmin performs a potential as a combinatorial anticancer drug for TNBC.

## Introduction

Chemotherapy remains the standard approach in most breast cancer patients, especially in the triple-negative breast cancer (TNBC) subtype. With the absence of estrogen receptor (ER)/progesterone receptor (PR) and human epidermal growth factor receptor 2 (HER2), this subtype of breast cancer is known to have the highest mortality rate (Kulkarni et al., 2019). Due to the lack of hormone targets, doxorubicin and cisplatin are the most often utilized chemotherapeutic treatments for TNBC therapy. Doxorubicin intercalates with DNA base pairs and causes DNA damage leading to the activation of various molecular signals to induce apoptosis (Tacar et al., 2012) as well as cisplatin, which causes DNA damage on cells by forming cross-linking covalent bonds on purine bases in 1,2-intrastrand DNA (Hu et al., 2016). Both chemotherapy drugs targeted DNA is also held by normal cells. As a result, normal cells are impacted by chemotherapy drugs’ side effects. In addition, cancer cells raise a resistance phenomenon to chemotherapeutic drugs such as doxorubicin (Dox) and cisplatin (Nedeljković and Damjanović, 2019). Therefore, developing new chemotherapeutic agents for TNBC treatment with low side effects and a high effectivity and selectivity is a great challenge.

We take the challenge by developing a new promising chemotherapeutic drug, namely PGV-1 (Pentagamavunon-1). PGV-1 is a curcumin analog that performs cytotoxic activities against cancer cells much better than curcumin by inhibiting the cell cycle in the G2/M phase, more precisely in the pro-metaphase lead to apoptosis (Lestari et al., 2019; Meiyanto et al., 2019). Moreover, PGV-1 on normal NIH3T3 cells does not cause cell death (Da’i et al., 2017), indicating that PGV-1 interacts with specific targets on cancer cells that are not affected in normal cells. Other studies show that PGV-1 inhibits matrix metalloproteinase 9 (MMP9) expression and cancer cell migration on 4T1 cells, indicating that PGV-1 can also be developed as an inhibitor of TNBC metastasis (Meiyanto et al., 2020). In vivo studies of PGV-1 on a mice xenograft model injected with K562 and 4T1 cells showed that PGV-1 is able to inhibit tumor formation with low side effects (Lestari et al., 2019; Meiyanto et al., 2020). Despite the high anticancer activity of PGV-1 on 4T1 cells indicated by a low IC_50 _value (4-10 μM) (Meiyanto et al., 2020), it is essential to enhance its effectiveness by combining it with other substances that are relatively not toxic, for example, citrus flavonoid (Meiyanto et al., 2012). On the other hand, the unique target mechanism of PGV-1 in inducing cell cycle arrest needs to be explored further to establish its specific molecular interaction, which is different from the normal cells, including the possibility of co-treatment with another compound.

Some natural products have been explored as adjunctive combinatorial treatments to increase chemotherapeutic drugs against 4T1 cells, such as galangal (Ahlina et al., 2020) and genistein (Ikawati et al., 2020). Here, we used diosmin, an abundant secondary metabolite in *Citrus sp. *(Chen et al., 2020), as a combinatorial agent candidate for PGV-1. Diosmin performs low to medium cytotoxic properties through inhibition of the cell cycle, induce senescence, apoptosis, and stimulate oxidative stress in breast cancer cells, namely MCF-7 (ER+, PR+/-, HER2-), MDA-MB-231 (ER-, PR-, HER2-), and SK-BR-3 (ER-, PR-, HER2+) (Lewinska et al., 2017; Meiyanto et al., 2012; Utomo et al., 2020). Besides, diosmin has also been widely used as a drug for hemorrhage (Sheikh et al., 2020) and supplement; therefore, that proves its safety. So far, the information concerning its molecular mechanism underlying the cytotoxic activities remains lack; thus, it is interesting to be identified further.

This study aims to observe the effect of the combinatory co-treatment of diosmin and PGV-1 in suppressing the growth of TNBC (4T1) cells to gain the increasing performance of PGV-1 as a cytotoxic agent against 4T1. This research was conducted using two approaches, namely in vitro and bioinformatic analysis. In vitro study focuses on the effect of cytotoxic activity, morphological changes in mitosis, and senescence acceleration of the diosmin and PGV-1 co-treatment in 4T1 cells. The bioinformatic analysis concerns the predictive output of diosmin and PGV-1 protein targets on overexpressed genes in TNBC, how they are expressed in various cancer cells, and targeted protein expression’s effect on patient survival. All the data can comprehend the potential application of combinatorial treatment of diosmin and PGV-1 to overcome the TNBC problems.

## Materials and Methods

*Compounds*

PGV-1 was obtained from Cancer Chemoprevention Research Center (CCRC), Faculty of Pharmacy, Universitas Gadjah Mada. Diosmin was purchased from Sigma Aldrich, USA.

*Cell culture*

Human breast cancer cell line 4T1 were cultured at 37°C in Dulbecco’s Modified Eagle’s Medium (DMEM) high glucose culture medium (Gibco, USA) containing 10% Fetal Bovine Serum (FBS) (Gibco, USA) and 1% penicillin-streptomycin (Gibco, USA) with a humidified atmosphere and 5% CO_2_ until confluence.

*Effects of PGV-1 and diosmin on cells growth of 4T1 cells*

The MTT assay was carried out based on Mosmann (1983) with modifications. A total of 5×10^3^ 4T1 cells were incubated with 10-500 µM diosmin or 0.5-16 µM PGV-1 and a combination of both for 24 hours. Cells were washed with PBS for each well as much as 100 µL 2-3 times, then added with MTT solution in the culture medium in each well and incubated for 2-3 hours. The MTT reaction was terminated by adding a stopper reagent (10% SDS in 0.01 N HCl). MTT plates were incubated overnight in dark conditions at room temperature. Furthermore, absorbance readings at a wavelength of 595 nm were carried out using a microplate reader. The absorbance data of single and combination treatments were then converted into percent cell viability to calculate the IC_50_ value in single treatment and combination index (CI) in the combination treatment of diosmin and PGV-1 (Zulfin et al., 2021).

*Effects on PGV-1 and diosmin on morphological changes in mitosis*

The cell cycle test with May-Grünwald-Giemsa staining is based on a previous assay conducted by Lestari et al., (2019). The procedure refers to the instructions given by the company Sigma Aldrich (USA) with modifications. 4T1 cells (density 1×10^5^ cells/well) were grown on 6-well plates, incubated for 24 hours, and grown to 80% confluent. After that, a single diosmin concentration series was made, and the combination with diosmin and PGV-1 was incubated for 24 hours. The staining was performed by washing cells with PBS and adding 200 µL of May-Grünwald stain solution to each well, incubated for 5 minutes, then discard the May-Grünwald stain. Following the addition of 200 µL of phosphate buffer, the plate was let to stand for 1.5 minutes, and the buffer was removed. The Giemsa solution, which has been diluted with 200 µL of aquabidest per well, was added, and the incubation was set at room temperature for 20 minutes, followed by solution removal and washing the cells with distilled water. The stained cells were dried and were observed by using an inverted microscope.

*Effects of PGV-1 and diosmin on inducing cell senescence*

The SA-β-galactosidase assay was conducted based as previously described (Debacq-Chainiaux et al., 2009). 4T1 cells (1×10^5^ cells/well) were grown on 6-well plates, incubated for 24 hours, and grown up to 80% confluent. After that, a single diosmin concentration series was made and combined with PGV-1 and incubated for 24 hours. The staining was done by removing the media from the plate and washing twice with PBS. The fixation buffer was added, incubated for 10-20 minutes, washed with PBS, added 1-2 mL X-Gal solution, then incubated in a non-CO_2_ incubator at 37°C. Cells were observed after 72 hours of staining with an inverted microscope. The senescence cells were quantified using ImageJ software carried out by two investigators independently, each calculating three fields of view to reduce the subjectivity.

*The targeted protein of PGV-1 and diosmin*

Bioinformatic studies were carried out on the SwissTargetPrediction website (http://www.swisstargetprediction.ch/). Protein expression was explored in the TCGA database using UALCAN platform (http://ualcan.path.uab.edu/), and for the Kaplan-Meier survival plot, we use Oncoln (http://www.oncolnc.org/) and utilizing GraphPad prism software V9.

*Molecular docking*

We performed molecular docking studies to confirm the binding relationship between PGV-1 and diosmin to CDK1, KIF11, and AURKA. This computational assay was arranged to simulate molecular binding, calculate RMSD, and view protein-ligand interaction, utilizing license-based software MOE 2010.10. The PDB ID were 6GU6, 3ZCW, and 2DWB for CDK1, KIF11, and AURKA, respectively. By employing the triangular match, London dG was the default setup and scoring method. The force field approach was utilized to refine the docking outcomes of at least ten holding settings.

In the ChemDraw program, the chemical structure of diosmin and PGV-1 was developed, and structural energy was reduced and produced for MOE’s conformational structure. The molecular docking investigation focused on the native linkage location of each protein. The molecular docking results described the affinity of every drug with the target proteins through the docking score and the link visualization.

*Statistical analysis*

Statistical significance was assessed using SPSS version 21.0. The One-Sample Kolmogorov-Smirnov Test was performed to determine the normality of the data. After confirming the data normality, the parametric test with one-way ANOVA using a post hoc test with Tukey HSD was carried out to determine the significance between groups with 95% confidence level (p ≤0.05). 

## Results

*Effects of PGV-1 and diosmin on cells growth of 4T1 cells*

**Figure 1:**
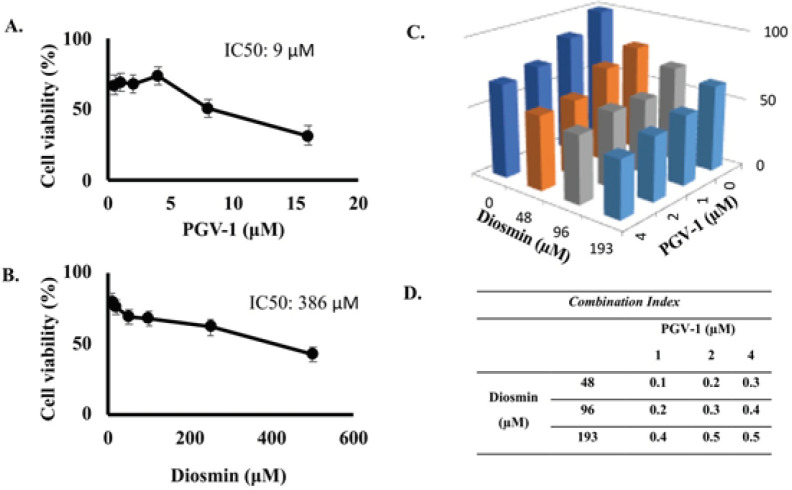
Cytotoxic Effects of PGV-1 and Diosmin on 4T1 Breast Cancer Cells. (A) Cytotoxic profile of PGV-1 treatment on 4T1 cell viability. (B) Cytotoxic profile of diosmin on 4T1 cells. Viable cells were counted according to the analysis procedure (p ≤0.05). (C) Cell viability of 4T1 with the treatment of diosmin sub-doses (48, 96, and 193 µM) combined with PGV-1 sub-doses (1, 2, and 4 µM). The combination treatment showed a decrease in cell viability compared to a single treatment of each compound. (D). A combination Index value of diosmin and PGV-1

To evaluate the cytotoxic effect of diosmin and PGV-1, we performed an MTT assay in the concentration range of 10-500 µM for diosmin and 0.5-16 μM for PGV-1 using 4T1 cells, which are known not to express ER/PR and HER2 (Kulkarni et al., 2019). The cytotoxic activity of PGV-1 was greater than that of diosmin, with an IC_50_ value of 9 µM for PGV-1 and 387 µM for diosmin (Fig. 1A and 1B). PGV-1’s high cytotoxic activity aligns with previous studies on various cancer cell lines (Meiyanto et al., 2020, 2019, 2014). After that, a combination cytotoxic test was carried out to evaluate the effect of diosmin on PGV-1 in inhibiting the proliferation of 4T1 cancer cells. The combined results showed that diosmin increased the cytotoxic effect of PGV-1 on 4T1 cells (Figure 1C). The combination of diosmin and PGV-1 showed a synergistic property with a combination index (CI) value of <1 (Figure 1D). The results of this synergistic combination indicate that diosmin increases the cytotoxic activity of PGV-1 against 4T1 cells.

*Effects of PGV-1 and diosmin on mitosis*

**Figure 2:**
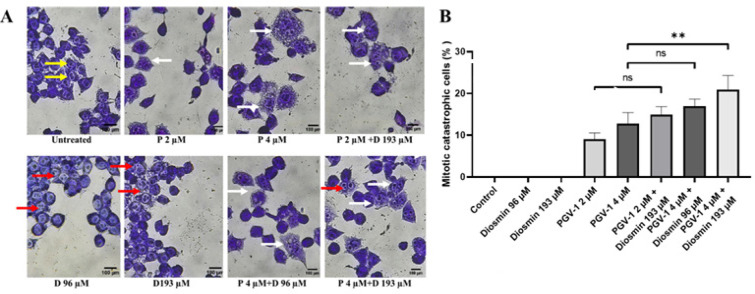
Mitotic Catastrophe Effects of PGV-1 (P) and Diosmin (D) Treatment on 4T1 Breast Cancer Cells. (A) 4T1 cells (1×10^5^ cells/mL) were treated with the PGV-1 sub-doses (4 and 9 μM), the diosmin sub-doses (96 and 193 μM) and the combination of both for 24 hours, then stained the cell nucleus using the May Grunwald-Giemsa reagent to see the mitotic catastrophe that occurs. Yellow arrows indicate normal cells, red arrows indicate G2/M arrest cells, and white arrows indicate mitotic catastrophes. (B) Quantification of cells undergoing mitotic catastrophes. ns (not significant): p > 0.05, **: p ≤ 0.01

The inhibitory activity of 4T1 cell proliferation is related to the inhibition of the cell cycle. The previous study has shown that PGV-1 acted in the G2/M phase to inhibit cell cycle progression, more precisely in the pro-metaphase in K562 cells (Lestari et al., 2019). We then observed whether or not PGV-1 in single or in co-treatment with diosmin affect mitosis progression in 4T1 cells by May-Grünwald-Giemsa staining. PGV-1 resulted in chromosome condensation but had not yet merged with the metaphase line and did not separate to the two-division poles so that the cell cycle stopped in the pro-metaphase. In addition, it is also seen that there are cells with catastrophic mitotic characteristics, namely the formation of giant cells with abnormal chromosomes (Vakifahmetoglu et al., 2008). This phenomenon was not seen in 4T1 cells with diosmin, but in the combination treatment of diosmin and PGV-1, a significant increase (p <0.01) in the number of cells that undergoing catastrophic mitotic and chromosome condensation could be seen (Figure 2A-2B). These results indicated that diosmin was able to increase the effectiveness of PGV-1 in inducing cell inhibition in the pro-metaphase phase and inducing the formation of cells with catastrophic mitotic characteristics.

*Effects of PGV-1 and diosmin on inducing cell senescence*

**Figure 3:**
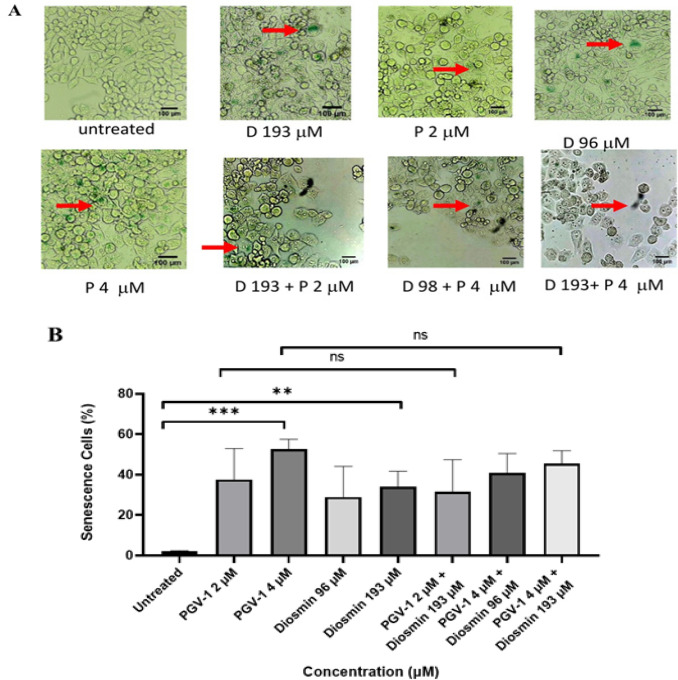
Effects of PGV-1 (P) and Diosmin (D) Treatment on 4T1 Senescent Cells. (A) Cells are experiencing senescence. The arrows show β-galactosidase-positive cells, which are expressed in senescent cells. (B) Quantification of 4T1 cells undergoing senescence. ns (not significant): p >0.05, **: p ≤0.01, ***: p ≤0.001

Inhibition in the G2/M phase and the formation of mitotic catastrophes can lead cells to senescence (Sikora et al., 2011). To obtain the cell senescence effect of PGV-1 and diosmin, we performed an SA-β-galactosidase assay. In this study, both PGV-1 and diosmin with single or combination treatments induced senescence in 4T1 cells (p<0.001) (Figure 3A-B). The combination with diosmin is known to induce senescence but did not show a significant difference compared to the single treatment of PGV-1 (p>0.05) (Figure 3A-B). This result follows previous studies where PGV-1 could induce senescence in K562 and 4T1 cells (Lestari et al., 2019; Meiyanto et al., 2020). Diosmin administration on MCF-7 and MDA-MB-231cells has also been shown to induce senescence (Lewinska et al., 2017). Interestingly, both PGV-1 and diosmin induce cell senescence on 4T1 cells.

*The targeted protein of PGV-1 and diosmin on overexpression gene of TNBC*

**Figure 4:**
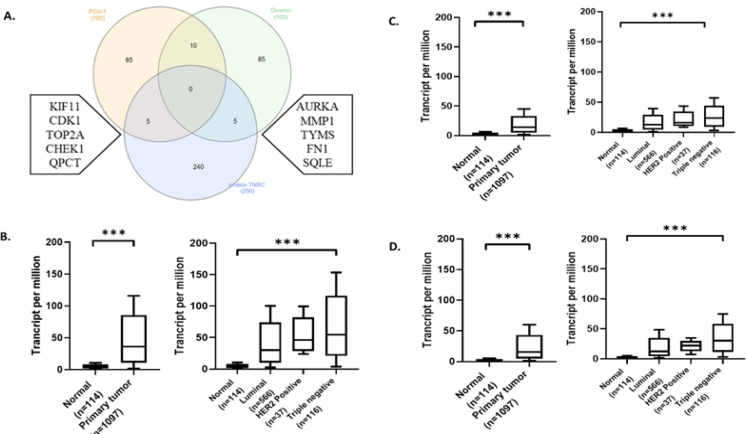
Venn Diagram of a Predictive Target Protein of PGV-1 and Diosmin with Protein Overexpression in TNBC Breast Cancer and Expression of Predicted Target Proteins in a Normal Cell, Cancer Cell, and Triple-Negative Breast Cell. (A) Predicted target proteins for PGV-1 and diosmin (B) CDK1 expression in normal cells, cancer cells, and various breast cancer. (C) Comparison of KIF11 expression on various types of cells and breast cancer cells. (D) Expression of AURKA on cell normal, cancer cell, and various breast cancer cells. This TCGA dataset is accessed via UALCAN (http://ualcan.path.uab.edu). ***: p ≤0.001

Mitotic catastrophe can be formed by the occurrence of deviations in the process of entering the mitotic phase. Therefore, the disruption of proteins that play a role in the completion of DNA replication can induce the formation of mitotic catastrophes (Castedo et al., 2004). Proteins that play a role in the mitosis entry process include CDK1, CycB, AURKA, AURKB, and APC (anaphase-promoting complex) proteins (Castedo et al., 2004; Ma et al., 2012). The exploration results through the SwissTargetPrediction (www.swisstargetprediction.ch) show that three target proteins for both compounds are obtained from the overexpressed proteins by TNBC breast cancer; three proteins are closely related to cell cycle regulation. The predictive targets for PGV-1 proteins are CDK1 and KIF11, with diosmin targeting AURKA (Figure 4A). These results further confirm the anticancer activity of the combination of diosmin and PGV-1 through the catastrophic mitotic mechanism.

*Expression of CDK1, AURKA, and KIF11 on TNBC cancer*

We further investigated the expression of predicted target proteins of PGV-1 and diosmin in TCGA database using UALCAN website. Figure 4B shows that the expression of CDK1 in cancer cells is higher than that of in normal cells; after further investigation, it was found that CDK1 expression among various breast cancers showed higher expression in breast cancer with TNBC subtype. Expression of KIF11 protein (Figure 4C) is higher in cancer cells than normal cells, and among breast cancers, KIF11 expression in TNBC sub-type breast cancers was also significantly higher (p ≤ 0.05). AURKA protein expression (Figure 4D) was higher in tumor cells than normal cells and higher in TNBC sub-type breast cancers compared to other breast cancers. The high expression of the three target proteins in tumor cells, compared to normal cells or in TNBC, suggests that these proteins have an essential role in cell cycle modulation in TNBC breast cancer. A further investigation was carried out to see the survival rate of patients with overexpression of CDK1, KIF11, and AURKA.

*The survival rate of breast cancer patients with overexpression of CDK1, AURKA, and KIF11*

**Figure 5:**
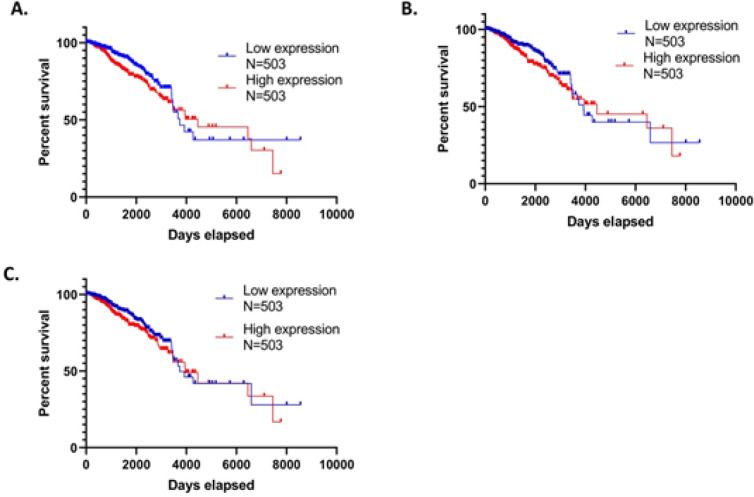
The Survival Rate of Patients with Over and Low Expression of Predicted Target Proteins in the Breast Cancer Patient. (A) The survival rate of patients with over and low expression of CDK1. p-value of 0.048 (B) Survival rate of patients with high and low expression of KIF11 with p-value of 0.141, and (C) patients with high and low expression of AURKA with a p-value of 0.129. This TCGA dataset is accessed via Oncoln (http://www.oncolnc.org/).

The overexpression of these three proteins will impact patients, so further investigation is needed regarding the impact of overexpression or low expression of these three proteins on the survival of breast cancer patients. The TCGA dataset was searched using the Oncoln website by entering the desired protein and then determining the number of samples for each patient with high and low expressed protein. The greater the number of samples included, the more significant the results obtained, so every 503 samples were selected for high and low expressions. Figure 5A shows the survival of samples with high (red line) and low (blue line) CDK1 expression. Sample with high CDK1 expression showed a lower percent survival and a faster lifespan than patients with low CDK1 expression. This result shows that the high expression of CDK1 in breast cancer patients influences patient survival.

The overexpression of KIF11 (Figure 5B) affected the survival of patients with breast cancer. Samples with high expression (red line) have lower percent survival and lifetime than samples with low KIF11 expression (blue line) likewise, with the overexpression of AURKA protein in breast cancer patients (Figure 5C). Samples with high AURKA expression (red line) have shorter survival and lifespan than samples with lower AURKA expression (blue line). These results indicate that the overexpression of the three protein targets of diosmin and PGV-1, namely CDK1, KIF11, and AURKA, has an adverse effect on the survival of breast cancer patients so that these three targets are essential in breast cancer, especially the TNBC type breast cancer that does not have an expression of HER, ER, and PR target receptors.

*Interaction of PGV-1 and diosmin on targeted proteins by molecular docking*

**Figure 6:**
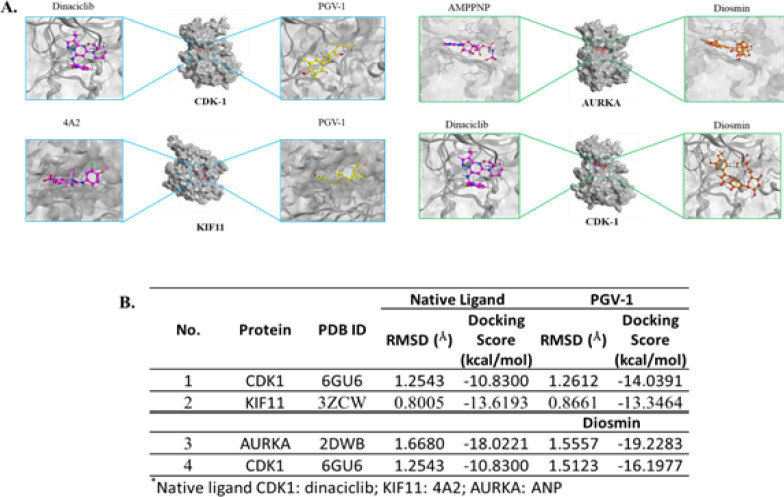
Docking Simulation of Molecular Interaction between the Ligands and the Respective Proteins. (A) Crystal structure of AURKA and CDK1 (shown in grey) and binding interaction model of its native ligand and diosmin (left panel). Crystal structure of KIF11 and CDK1(shown in grey) and binding interaction model of its native ligand and diosmin (right panel). Docking score of ligands toward AURKA and CDK1 and KIF11. (B) RMSD and docking score of PGV-1 and diosmin with the protein target

We then performed molecular docking under MOE software to confirm the interaction between PGV-1 or diosmin and the protein target predictions. In this concern, we simulated protein targets CDK1 and KIF11 for PVG-1, while CDK1 and AURKA as the protein targets for diosmin. Those protein targets are recognized as the most preferable protein targets based on bioinformatic analysis (Figure 6A). The docking results showed that all those interactions exhibit interatomic distance, which is valid to be analyzed (RMSD <2Å). PGV-1 performed a lower docking score (-14.0391 kcal/mol) to CDK1 than that of the native ligand (-10.8300 kcal/mol), indicating a higher binding affinity PGV-1 to CDK1.

Meanwhile, the docking score of PGV-1 to KIF11 (-13.3464 kcal/mol) is relatively the same as that of the native ligand for KIF11 (-13.6193 kcal/mol) that show they have the same binding affinity. In addition, based on the docking scores, we found that diosmin performed better affinity compared to the native ligand against both AURKA and CDK1 (Figure 6B). Thus, we verify that mitotic regulator proteins are the target of PGV-1 and diosmin in inducing cell cycle arrest and mitotic catastrophe. 

**Figure 7:**
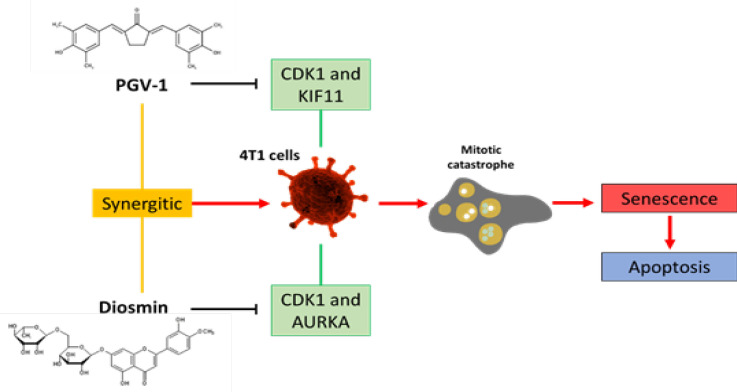
Graphical Abstract of how PGV-1 and Diosmin were Causing Inhibition in the Cell Cycle and Induce Mitotic Catastrophe on 4T1 Cancer Cells, Thereby Causing Cells to Undergo Apoptosis by Targeting the Cell Cycle Proteins CDK1, KIF11, and AURKA

## Discussion

The PGV-1 (a curcumin analogue) is a promising candidate for anticancer drugs, especially for TNBC. This investigation focuses on determining its mechanism on mitosis abrogation effect in a single and combinatorial treatment with diosmin. Fortunately, diosmin gives a synergistic effect with PGV-1 in suppressing the cell growth of 4T1 cells. Hence, PGV-1 and diosmin are not toxic to normal tissues in vitro and in vivo, so we expect those combination treatments would not affect non-cancerous cells. Nevertheless, we still should consider the possible unexpected effect due to the long-term application of that combination that should be explored further. 

The increasing effect of diosmin in mitotic catastrophe by PGV-1 exerts a new insight mechanism of those killing the cells through mitotic arrest. Moreover, this mitotic catastrophe effect seems to be correlated with the senescent induction effect that commonly occurs in proliferating cells such as cancer cells (Ou et al., 2020). In this case, we used 4T1 cells characterized as actively proliferating cancer cells with a doubling time of about 14 hours (Simoes et al., 2014). This phenomenon indicates that PGV-1 and its combination with diosmin are suitable for TNBC with active proliferation and are expected not to raise adverse effects. Our bioinformatics analysis may confirm those claims. Among the G2/M protein regulators, PGV-1 targets CDK1 and KIF11, while diosmin targets AURKA. All of those proteins are overexpressed in TNBC, as well as in 4T1 cells, and give as important protein markers that affect the survival rate of patients. Therefore, targeting those proteins is markedly essential to distinguish them from the normal tissues. 

The three protein targets of PGV-1 and diosmin possess essential roles in mitosis progression. CDK1 controls mitosis progression through prophase until anaphase through Mitotic Promoting Complex (MPC) formation with cyclin-B (Izadi et al., 2020; Shah et al., 2020). Inhibiting MPC disrupts cell cycle progression and resulting in cell cycle arrest in prophase, pro-metaphase, metaphase, or anaphase (Raab et al., 2019). KIF11 belongs to the kinase protein regulating spindle bipolar conformation during pro-metaphase to metaphase (Pei et al., 2017). The inhibition of KIF11 activity correlates to the inhibition of spindle formation that abrogates chromatin arrangement in the equatorial (Venere et al., 2015). In this regard, we confirmed that PGV-1 interacts finely with CDK1 and KIF11, giving insight into the underlying mechanism of PGV-1 to induce cell cycle arrest in pro-metaphase. Our finding also provides additional information that diosmin contribute to the inhibitory effect of PGV-1 on mitotic arrest targeting on AURKA, besides CDK1. AURKA regulates maturation and separation of the centrosome, followed by the conformation of spindle bipolar, hence triggering mitotic entry (Du et al., 2021; Dutertre et al., 2004; Liu and Ruderman, 2006; Mahen and Venkitaraman, 2012). This additional inhibition of protein regulators in the early stage of mitosis may trigger mitotic catastrophe and cell senescence due to the abrogation of chromatin arrangement. Mitotic catastrophe can be formed by the occurrence of deviations in the process of entering the mitotic phase (Figure 7). So the disruption of proteins that play a role in completing DNA replication can induce the formation of mitotic catastrophes that may continue to be apoptosis (Castedo et al., 2004). 

This study provides additional information that PGV-1 is the potential to be developed as a chemotherapeutic agent. Previous studies have mentioned many advantages of PGV-1 compared to curcumin in its anticancer activity (Lestari et al., 2019; Meiyanto et al., 2019). In addition, in vivo experiment on mouse models injected with 4T1 and K562 cells showed that oral administration of PGV-1 was able to inhibit tumour formation with low side effects, characterized by slight weight loss and no reduction in red blood cell counts and white blood cells and there was no effect on behavior and appearance (Lestari et al., 2019; Meiyanto et al., 2020). On the other hand, the safety of the use of diosmin is known by its widespread use as hemorrhoid drugs or venaroid drugs (Daflon 500^®^, ANADIUM^®^, ARDIUM^®^) and health supplements (DiosVein^®^). This study shows that the combination of diosmin with PGV-1 can improve the performance of PGV-1 as a chemotherapeutic agent; however, further investigation is needed to analyze the molecular mechanism and in vivo study of the combination of these two compounds so that the development of the combination of these two compounds as an orally-administrated cancer drug.

In conclusion, PGV-1 induces mitotic arrest in 4T1 cells with mitotic catastrophe characteristics and correlates with senescence evidence. Diosmin enhances the cytotoxic effect of PGV-1 on 4T1 cells in correlation with a catastrophic event in mitosis that eventually increases senescence. The synergistic effect of PGV-1 and diosmin may be mediated through the inhibition of CDK1 and KIF11 by PGV-1 and AURKA and CDK1 by diosmin. Diosmin and PGV-1 can be developed to be a co-chemotherapeutic agents for TNBC.

*Abbreviations*

SA-ß-gal: SA-β-Galactosidase Assay

CDK1: cyclin dependant kinase 1

KIF11: kinesin family member 11

AURKA: aurora-A kinase

AURKB: Aurora-B kinase

CycB: cyclin B

PGV-1: Pentagamavunon-1

MTT: 3-(4,5-Dimethylthiazol-2-yl)-2,5-diphenyltetrazolium bromide

TNBC: Triple Negative Breast Cancer

RMSD: root-mean-square deviation

TCGA: the cancer genome atlas

## Author Contribution Statement

HM: performing most of the experiments, data collection and mining, analysis, and drafting of the original manuscript. AFN: performing the data mining and analysis of bioinformatic studies. ADA: data analysis. FW: performing molecular docking experiments and analysis. MI: supervising and manuscript preparation. EM: study design, supervised all the experiments, manuscript preparation, and giving the final approval of the manuscript. All authors contributed in manuscript revision. 
